# A Zebrafish Acromegaly Model Elevates DNA Damage and Impairs DNA Repair Pathways

**DOI:** 10.3390/biology7040047

**Published:** 2018-10-17

**Authors:** Abdalla Elbialy, Shuichi Asakawa, Shugo Watabe, Shigeharu Kinoshita

**Affiliations:** 1 Graduate School of Agricultural and Life Sciences, The University of Tokyo, Bunkyo, Tokyo 113-8657, Japan; abdallakhiry@gmail.com (A.E.); asakawa@mail.ecc.u-tokyo.ac.jp (S.A.); 2 Faculty of Veterinary Medicine, Damanhour University, Damanhour 22511, Egypt; 3 School of Marine Biosciences, Kitasato University, Minami, Sagamihara, Kanagawa 252-0313, Japan; swatabe@kitasato-u.ac.jp

**Keywords:** growth hormone, acromegaly, DNA damage, DNA repair

## Abstract

Acromegaly is a pathological condition due to excess growth hormone (GH) secretion. Acromegaly patients exhibit a deterioration of health and many associated complications, such as cardiovascular issues, arthritis, kidney diseases, muscular weakness, and colon cancer. Since these complications are generalized throughout the body, we investigated the effect of GH excess on cellular integrity. Here, we established stable acromegaly model zebrafish lines that overexpress tilapia GH and the red fluorescence protein (RFP) reporter gene for tracking GH gene expression throughout generations, and performed RNA-Seq data analysis from different organs. Intriguingly, heatmap and Expression2Kinases (X2K) analysis revealed the enrichment of DNA damage markers in various organs. Moreover, H2A.X immunostaining analysis in acromegaly zebrafish larvae and the adult acromegaly model brain and muscle showed a robust increase in the number of DNA-damaged cells. Using Gene Set Enrichment Analysis (GSEA), we found that the acromegaly zebrafish model had impaired DNA repair pathways in the liver, such as double-strand break (DSB), homologous recombination repair (HRR), non-homologous end joining (NHEJ), nucleotide excision repair (NER), and translesion synthesis (TLS). Interestingly, the impairment of DNA repair was even more prominent in acromegaly model than in aged zebrafish (three years old). Thus, our study demonstrates that affection of cellular integrity is characteristic of acromegaly.

## 1. Introduction

Acromegaly is a hormonal disorder and pathological condition predominantly caused by a growth hormone (GH)-secreting pituitary adenoma. Acromegaly patients show abnormal overgrowth accompanied by severe health deterioration and pathologies, such as neuropathies, kidney disease, heart diseases, diabetes mellitus, rheumatoid arthritis, an increased incidence of colon cancer, and high mortality [1,2,3]. However, the means by which GH excess induces these complications remains to be clarified.

Growth is a multifactorial, complex process in which GH plays a central role in all vertebrates. GH is synthesized and secreted by somatotrophs in the anterior pituitary. GH secretion is a multifactorial process that results from the interaction of many factors under both positive and negative feedback, which adjust the secretion according to body needs [4]. Major regulatory factors include GH-releasing hormone (GHRH), somatostatin, GH-releasing peptide (ghrerin), insulin-like growth factor 1 (IGF1), and GH itself. The principal physiological regulation mechanisms of GH secretion are neural endogenous rhythm, sleep, stress, exercise, and nutritional and metabolic signals [5]. Body growth is regulated by the GH/IGF axis, which is also involved in aging and age-related diseases [6]. Key downstream networks of the GH/IGF axis include the PI3K–AKT–TOR pathway and the RAF–MAPK pathway, which regulate cellular growth, apoptosis, proliferation, and aging [7].

Reduced IGF-I signaling in mice is associated with many health benefits [8], such as delayed aging and markedly extended longevity and immunity against cancer and diabetes mellitus. Although the lifespan of most normal mice is about two years, hypopituitary and GH-resistant mutants often survive beyond the age of three years and occasionally past the age of four years [8]. In humans, Laron dwarfism (GH receptor deficiency) is associated with a major reduction in pro-aging signaling, cancer, and diabetes [9]. On the other hand, GH excess in acromegaly is associated with health deterioration and many associated complications, which are generalized throughout the body. This prompted us to investigate whether GH excess in acromegaly affects cellular integrity.

Here, we produced an acromegaly zebrafish (*Danio rerio*) model overexpressing tilapia (*Oreochromis mossambicus*) GH and analyzed the DNA repair signaling pathways and DNA damage using different approaches.

## 2. Materials and Methods

### 2.1. Transgene Construction

**Figure 1:**
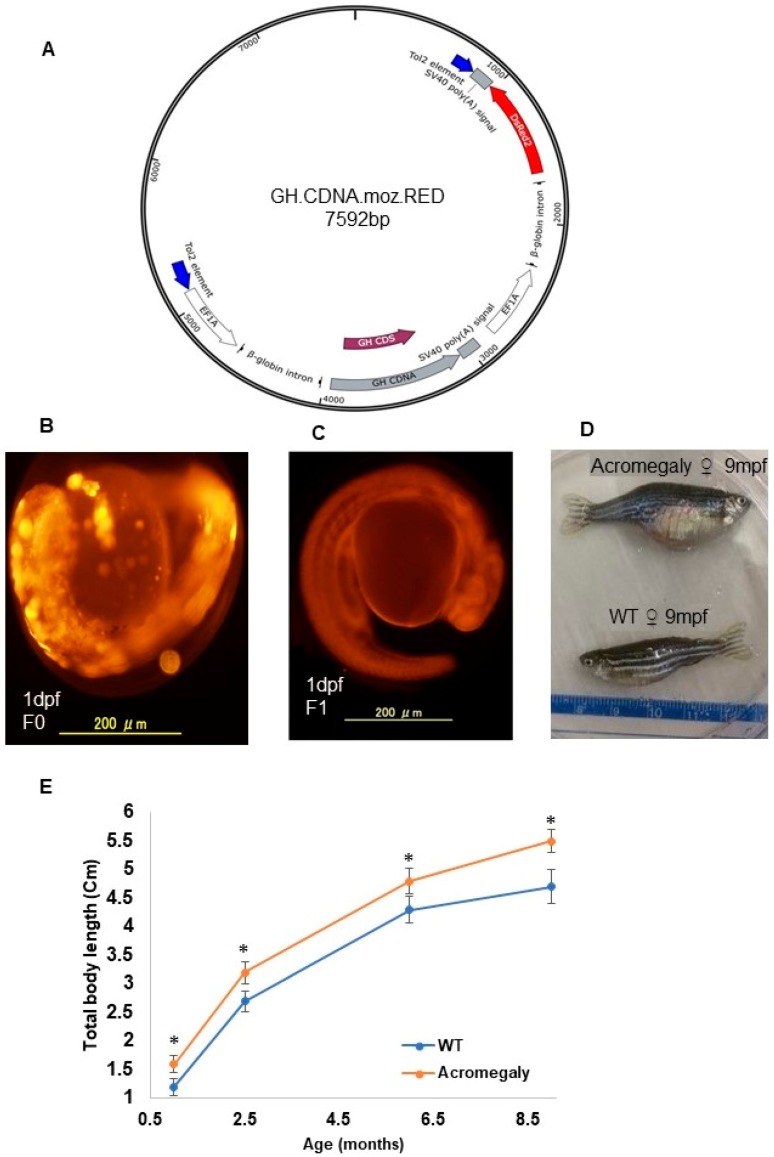
Production of the acromegaly zebrafish model overexpressing tilapia GH. (**A**) The structure of the GH-CDNA-moz-RED construct used to produce acromegaly zebrafish. The blue arrows indicate Tol2 transposable element. EF1A promoters drive expression of Tilapia GH cDNA and DsRed2 CDS. (**B**) Acromegaly zebrafish F0 generation larvae (1 dpf) showing mosaic expression of red fluorescent protein (RFP) reporter gene expression. (**C**) Acromegaly zebrafish F1 generation larvae (1 dpf) showing homogenous expression of red fluorescent protein (RFP) reporter gene expression in the whole body. (**D**) Representative female acromegaly zebrafish F1 generation (9 mpf) showing a progressive increase in growth rate compared to wild-type (WT). (**E**) Shown are growth curves represented as the total body length of fish recorded from one month till nine months of age between eight F1 Acromegaly zebrafish (four males and four females) and eight WT zebrafish (four males and four females). Data are expressed as the mean ± SE (*n* = 8). Statistical differences (*t*-test, *P* < 0.05) are denoted by asterisks. dpf: days post-fertilization, mpf: months post-fertilization.

All experimental procedures were approved by the Institutional Animal Care and ethics committee of Tokyo University. Ethical code: P14-952. Tilapia GH cDNA (GenBank Accession Number Y11732.1) was amplified using tilapia pituitary cDNA as a template. Using infusion cloning PCR (Takara, Tokyo, Japan; Cat no: # Z9648N), the PCR product was cloned into the pT2AL200R150G vector [10] to produce the GH.CDNA.moz.RED construct (Figure 1A), which is comprised of two copies of the elongation factor 1 alpha (EF1) promoter. One copy drives the expression of GH cDNA and the second copy drives the expression of the red fluorescence protein (RFP) reporter gene (Figure 1A).

### 2.2. Production of the Acromegaly Zebrafish First Generation (F0)

The constructed plasmid was introduced into One Shot TOP10 chemically competent *Escherichia coli* (Invitrogen, Carlsbad, CA, USA), cultured, and prepared using the Qiagen Plasmid mini kit (Qiagen, Duesseldorf, Germany). The Tol2 transposon system [11] was used to increase the plasmid integration frequency. The transposase mRNA was prepared from the pCS-zT2TP [12] plasmid using the mMessage mMachine Sp6 Kit (Ambion, Austin, TX, USA). The Qiagen RNeasy Kit (Qiagen) was used to purify the RNA solution.

We produced the acromegaly first generation (F0) zebrafish by microinjection of the construct into one-cell stage zebrafish embryos together with transposase mRNA (Figure 1B).

### 2.3. Production of the Acromegaly Zebrafish Second Generation (F1)

A group of embryos (F0) showing strong RFP expression were selected for rearing until sexual maturity. Five sexually mature female and four male acromegaly zebrafish (F0) were mated with wild-type fish to produce a heterogeneous F1 generation, which also expressed RFP. Unlike the F0 generation, which expressed RFP in mosaic form (Figure 1B), the F1 generation showed a homogenous expression of RFP (Figure 1C). We established stable acromegaly model zebrafish lines. We performed the following experiments using F1 acromegaly model zebrafish expressing RFP. For the calculation of growth curves in the F1 acromegaly model compared to WT zebrafish, we measured the total body length, including the caudal fin.

### 2.4. Reverse Transcription Polymerase Chain Reaction (RT-PCR)

The total RNAs from wild-type (WT) and Acromegaly model zebrafish larvae (three days post-fertilization (dpf)) were extracted by the RNeasy mini kit (Qiagen, Hilden, Germany), including DNase treatment according to the manufacturer’s instructions to remove all traces of DNA. RNA from the muscle, brain, and liver of the acromegaly model and WT (one-year-old) was isolated the same way. The PrimeScript™ II 1st strand cDNA Synthesis Kit (Takara; Cat no: #6210A) was used to synthesize cDNA. A reverse transcription polymerase chain reaction (RT-PCR) was performed using GH. F and GH. R primers (Table S1) for the detection of tilapia growth hormone gene expression.

### 2.5. Quantitative PCR (qPCR)

Acromegaly model and wild-type (WT) zebrafish larvae (three days post-fertilization (dpf)) were collected. qPCR was performed in triplicate. The total RNAs from 25 embryos were purified using Trizol reagents (Invitrogen), and the PrimeScript™ II 1st strand cDNA Synthesis Kit (Takara; Cat no: #6210A) was used to synthesize cDNA. RNA from the muscle, brain, kidney, and liver of the acromegaly model and WT (1-year-old) was isolated the same way and qPCR was performed in triplicate as well. The housekeeping gene elongation factor 1 alpha (EF1) was used as an internal control. The used genes and primer sequences are listed in Table S2. All reactions were performed in a 20 µL volume, containing 10 µL of SYBR Premix Ex Taq (Tli RNaseH Plus) (Takara), 0.4 µL of each primer (10 µM), 2 µL cDNA (50 ng), and 0.4 µL ROX Reference Dye. Thermal cycling was performed under the following conditions: 2 min at 95 °C, 15 s at 95 °C, 15 s at 58 °C, and 26 s at 72 °C, with a final extension step for 5 min at 72 °C. Data were collected using a 7500 real-time PCR system (Applied Biosystems, Foster City, CA, USA).

### 2.6. RNA-Seq and Data Analysis

#### RNA Isolation and Library Construction

The total RNAs from the acromegaly model and WT larvae (3 dpf) were purified using Trizol reagents (Invitrogen) according to the manufacturer’s instructions. Additionally, RNA from the brain, kidney, and liver of the acromegaly model, WT (one-year-old), and aged zebrafish (three-year-old) was isolated by Trizol. RNA quantity and quality were assessed using the Qubit RNA Assay Kit using a Qubit 2.0 fluorometer (Life Technologies, Gaithersburg, MD, USA) and the 2100 Bioanalyzer (Agilent Technologies, Palo Alto, CA, USA), respectively. The TruSeq standard mRNA Sample Prep Kit (Illumina, San Diego, CA, USA) was used for the generation of the cDNA library for Illumina sequencing. RNA sequencing was performed on the Illumina HiSeq 2500 platform (Illumina).

### 2.7. Mapping and Identification of Differential Gene Expression

Mapping of raw reads from acromegaly and WT samples to the zebrafish genome reference (GRCz10) retrieved from the Ensembl database (http://www.ensembl.org) was performed using HISAT2 software (http://ccb.jhu.edu/software/hisat2) according to the instructions from the program designers. The Cufflinks pipeline (http://cole-trapnell-lab.github.io/cufflinks/) was used to identify the relative transcript abundance (fragments of reads per kilobase of exon sequence per million mapped sequence reads (FPKM)) between the acromegaly model zebrafish and the WT samples.

### 2.8. Expression2Kinases (X2K)

Firstly, BioMart [13] was used to convert differentially expressed genes (DEGs) of acromegaly zebrafish to their human orthologues. Then, we used X2K [14] to identify the main kinases and transcription factors that regulate gene expression changes.

### 2.9. Heatmaps

The cummeRbund package of R software (https://bioconductor.org/packages/release/bioc/html/cummeRbund.html) was used to generate colored heatmaps from acromegaly zebrafish compared to the WT data.

### 2.10. Gene Set Enrichment Analysis (GSEA)

Using our RNA-Seq data, we performed GSEA [15] by first converting acromegaly zebrafish DEGs to their human orthologues using BioMart (http://www.ensembl.org/biomart/martview/cadc2978fa087d3abda8ff67cc386f83) and then testing for enrichment against double-strand break (DSB), homologous recombination repair (HRR), non-homologous end joining (NHEJ), nucleotide excision repair (NER), and translesion synthesis (TLS) gene sets [16]. For the detection of DNA damage, the gene set [17] in Table S3 was used. Empirical p-values were calculated by performing a bootstrap test in which the gene labels were shuffled 1000 times.

### 2.11. Isolation of Live Muscle and Brain Cells by Fluorescence-Activated Cell Sorting (FACS)

The acromegaly model and WT zebrafish (1-year-old) were euthanized in ice-cold water for 2 min, and then immersed in 70% (*v*/*v*) ethanol for skin sterilization. Zebrafish brain dissection was conducted as follows: in 400 µL of Fetal bovine serum (FBS), the brains collected from three fish from each group were disaggregated by repeated trituration with a 1000 µL tip and filtered through a 40 µm EASYstrainer (VWR, Seattle, WA, USA) for cell isolation. Following the addition of four volumes of Hank’s balanced salt solution (HBSS) (ThermoFisher Scientific, San Jose, CA, USA)/2% FBS, the samples were then washed and resuspended at a concentration of 10^6^/mL in phosphate-buffered saline (PBS)/30% FBS. Muscle dissection was conducted as described previously [18], with some modifications. First, 200 mg of muscle from each fish was mechanically disaggregated and incubated in DMEM medium containing 0.2% collagenase (Worthington Biochemical Corporation, Lakewood, NJ, USA) at room temperature with agitation for 60 min, followed by incubation with 0.006% trypsin at room temperature with agitation for 20 min. Cells were filtered through a 40 µm EASY strainer, and were then washed and resuspended at a concentration of 10^7^/mL in PBS/30% FBS containing 2 μg/mL propidium iodide (Sigma-Aldrich Japan, Tokyo, Japan) and kept on ice until analysis. FACS analysis was performed using an FACS Aria II (Becton Dickinson, San Jose, CA, USA). Propidium iodide fluorescence was excited by a 488 nm laser and detected using a 630/22 bandpass filter. Propidium iodide-positive dead cells were excluded. Only live cells were isolated for immunostaining.

### 2.12. Immunocytochemistry of FACS-Isolated Live Cells

FACS-isolated cells were fixed in PBS/4% paraformaldehyde (PFA) (Sigma-Aldrich Japan) for 10 min at room temperature, then washed in PBS, and smeared on gelatin-coated slides. After air-drying, the slides were rinsed with water to remove salt crystals. They were then permeabilized in PBS/0.2% Triton X-100 (Sigma-Aldrich Japan) for 20 min and blocked in PBS/1% bovine serum albumin (BSA) for 1 h at room temperature. Following this, the slides were incubated in PBS/1% BSA with rabbit H2A.X (phospho S139) antibody (Abcam, Cambridge, MA, USA; cat No.# ab81299) overnight at 4 °C. Slides were then washed five times with PBS (5 min each wash) and incubated for 1 h at 37 °C in PBS/1% BSA with Goat anti-Rabbit IgG (Alexa Fluor 488) (Thermo Fisher Scientific; cat. No. A-11034). Slides were again washed five times with PBS, for a total time of 5 min per wash. Slides were finally incubated with 4′,6-diamidino-2-phenylindole (DAPI) and mounted using Fluoromount™ Aqueous Mounting Medium (Sigma-Aldrich Japan; cat. No. F4680). Cells were imaged on a confocal laser scanning biological microscope (Olympus, Tokyo, Japan; cat. No. FV1200 BX61), and positive and negative H2A.X stained cells were also counted, before being classified into three classes: negative (nucleus negatively stained), grade I (H2A.X foci staining part of the nucleus), and grade II (H2A.X foci all over the nucleus).

### 2.13. Whole-Mount Immunostaining

The acromegaly model and WT zebrafish larvae (9 dpf) were washed with PBS, and then fixed in 4% paraformaldehyde (PFA) overnight at 4 °C. The larvae were treated with methanol for 2 h at −20 °C to enhance permeabilization, and then washed three times in PDT (0.1% Tween-20, 0.3% Triton-X, 1% dimethyl sulfoxide (DMSO) in PBS) with gentle shaking, with a total time of 30 min per wash. The larvae were incubated in blocking solution with 5% BSA for 1 h at room temperature with gentle shaking prior to overnight incubation with rabbit H2A.X (phospho S139) antibody (Abcam; cat No.# ab81299) at a 1/350 dilution in blocking solution. To remove residual primary antibody, larvae were sequentially washed three times with PDT (30 min each wash) and then incubated again in blocking solution with 5% BSA in PBST for 1 h at room temperature with gentle shaking prior to incubation with anti-rabbit, Alexa Fluor 488 (ThermoFisher Scientific; cat. No. A-11034) antibody at a 1/150 dilution in the dark for 2 h at room temperature, and then washed three times in PDT for 30 min each. For counterstaining, larvae were incubated with DAPI at 5/250 dilution in the dark, and then stored in blocking buffer at −4 °C until examination.

### 2.14. Acridine Orange Staining

The acromegaly model and WT embryos (3 dpf) were washed twice with PBS and incubated in PBS containing 2 µg/mL acridine orange (Sigma-Aldrich Japan; cat. No. 318337) for 30 min at 28 °C. Then, embryos were washed five times with E3 buffer. Tricaine-tranquilized embryos were mounted in 4% methylcellulose (Sigma-Aldrich Japan; cat. No. M0387) and visualized using (Olympus; cat. No. FV1200 BX61). Fiji software was used for the quantification of the apoptotic cell number.

### 2.15. Data Availability

The data that support the findings were submitted to Gene Expression Omnibus, under the accession number GSE113169.

## 3. Results

### 3.1. Tilapia GH is Physiologically Active in the Acromegaly Zebrafish Model

To produce a zebrafish acromegaly model, we performed the overexpression of tilapia GH instead of zebrafish *gh1* to be able to differentiate between endogenous and exogenous GH gene expression. Additionally, previous studies showed that tilapia GH can accelerate growth in transgenic zebrafish [19]. Similarly, the established stable acromegaly model zebrafish lines that coexpressed red fluorescent protein (FRP) showed significant enhancement of the growth rate (Figure 1D,E).

**Figure 2:**
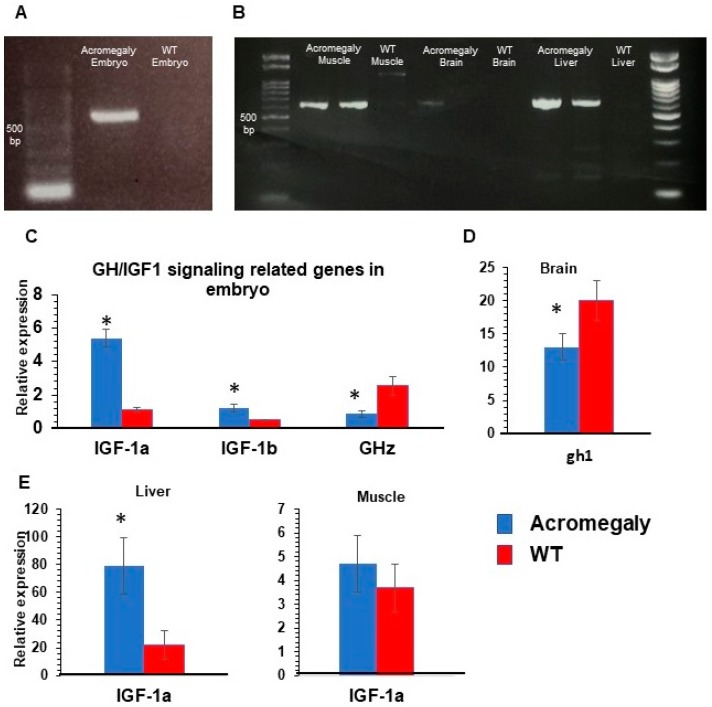
GH/IGF1 signaling-related gene expression in Acromegaly zebrafish. (**A**,**B**) RT-PCR showing tilapia GH gene expression in acromegaly zebrafish larvae (3 dpf) (**A**), acromegaly muscle, and acromegaly liver (-year-old), (**B**) but not in wild-type (WT). (**C**–**E**) Relative gene expression of GH/IGF1 signaling pathway-related genes from acromegaly zebrafish at larval stage (3 dpf) (**C**), one-year-old acromegaly Brain (**D**), and acromegaly muscle and liver (**E**), versus wild-type (WT). IGF1a (insulin-like growth factor 1), IGF1b (insulin-like growth factor 3), and GHz (zebrafish growth hormone). Data are expressed as the mean ± SE (*n* = 6). Statistical differences (*t*-test, *P* < 0.05) are denoted by asterisks. dpf: days post-fertilization, mpf: months post-fertilization.

Then, we wanted to investigate whether tilapia GH was expressed in our acromegaly zebrafish model. Tilapia GH gene expression was confirmed by reverse transcriptase PCR in the first-generation (F1) three days post-fertilization (3 dpf) larvae, and F1 one-year-old acromegaly muscle and liver (Table S1 and Figure 2A,B), but not in WT larvae or one-year-old WT fish. On the other hand, endogenous zebrafish *gh1* gene expression was significantly reduced in acromegaly larvae and one-year-old acromegaly brain (including pituitary gland) (Figure 2C,D), likely due to a negative feedback loop involving GH excess [20]. Using qPCR, we did not detect endogenous zebrafish gh1 in other organs in WT or the acromegaly model.

GH induces the secretion of IGF1, mainly from the liver. IGF1 usually mediates GH physiological growth effects [21]. In agreement with this, the qPCR results showed a significant induction of the expression level of IGF1a (5-fold) and IGF1b (Table S2 and Figure 2C) in the acromegaly model (F1) larvae and adult acromegaly liver (one-year-old), but not in muscle (Figure 2E). Taken together, these observations indicate that tilapia GH is physiologically active in the acromegaly zebrafish.

### 3.2. The Acromegaly Zebrafish Have Elevated DNA Damage

To investigate whether GH excess affects cellular integrity, we focused on genomic instability, DNA damage, and repair. Genome instability is caused by a high frequency of mutations within the cell genome. These mutations can involve changes in nucleic acid chromosomal rearrangements. Genome instability is central to carcinogenesis, aging, and the pathogenesis of neurodegenerative diseases. The main source of genome instability is increased DNA damage [22].

**Figure 3:**
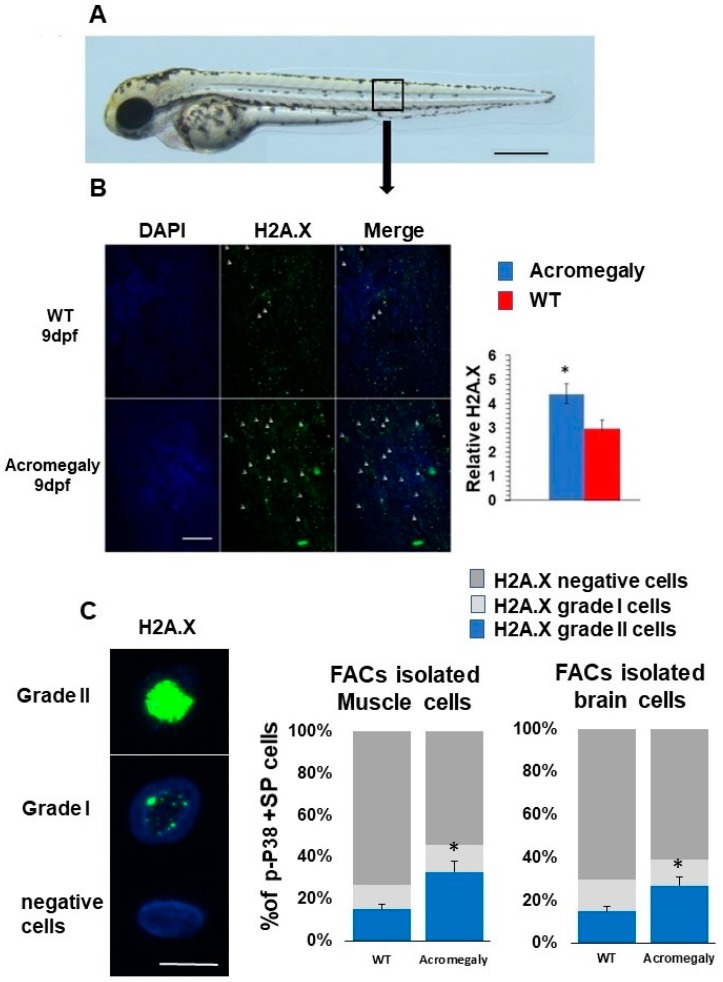
Increased DNA damage in Acromegaly zebrafish larvae and adult. (**A**) A zebrafish larva showing the area for taking the image for H2A.X quantification (black box). Bar = 500 µm. (**B**) Representative H2A.X whole-mount immunostaining and quantification in acromegaly and WT zebrafish (9 dpf) showing a significant increase in the number of H2A.X stained cell nuclei in the acromegaly zebrafish model. Arrows in panel B show H2A.X stained cell nuclei. Bar = 40 µM. Quantification was performed using Fiji software. (**C**) Representative H2A.X grade I, grade II, and negatively stained cells. Bar = 5 µM (left). Quantification of H2A.X positive and negative stained live isolated cells from the muscle and brain of acromegaly and WT (one-year-old) fish (left). Data are expressed as the mean ± SE (*n* = 3). Statistical difference (*t*-test, *P* < 0.05) is denoted by an asterisk.

**Figure 4:**
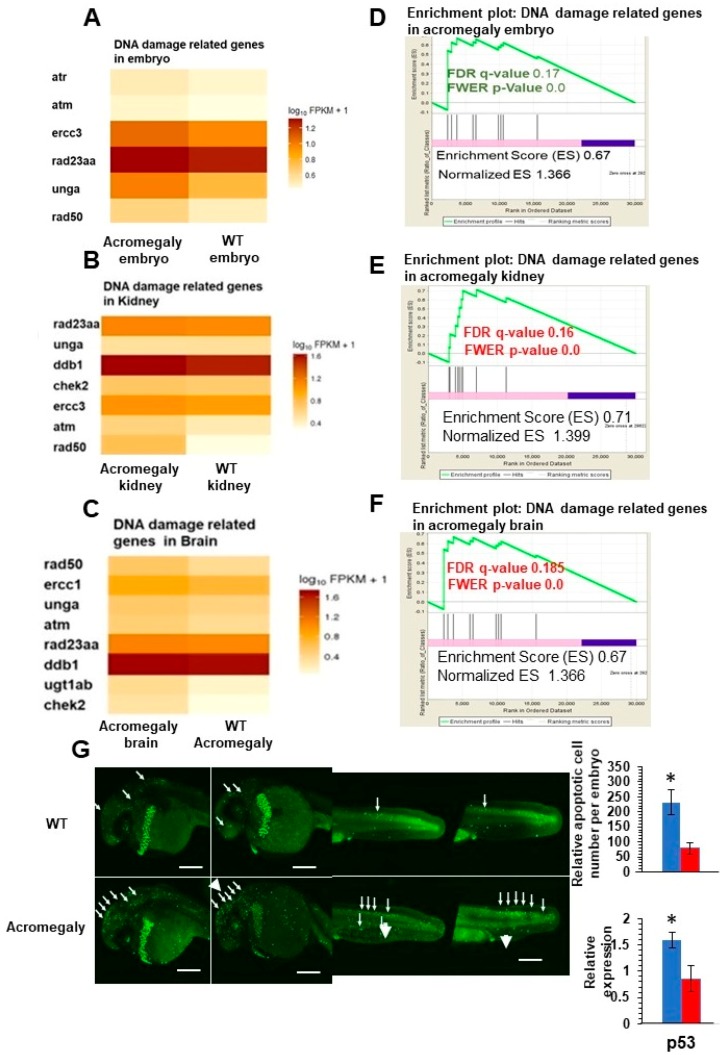
Increased DNA damage in various organs of acromegaly zebrafish model. (**A**–**C**) Heatmap of DNA damage markers in acromegaly larvae (3 dpf) (**A**), acromegaly kidney (one-year-old) (**B**), and acromegaly brain (one-year-old) (**C**) versus WT. (**D**–**F**) The results of GSEA showing the familywise-error rate (FWER) *p*-values, false discovery rate (FDR), enrichment scores (ES), and normalized ES of DNA damage markers in acromegaly larvae (3 dpf) (**D**), acromegaly kidney (one-year-old) (**E**), and acromegaly brain (one-year-old) (**F**) versus WT. Significant *p*-value < 0.05 and FDR *q*-value < 0.25 are written in red. According to GSEA [15], the reported *p*-value of 0.0 indicates an actual *p*-value of less than 0.01 (**G**) Representative Acridine orange (AO) staining and quantification of acromegaly model and WT larvae. The quantification of AO positive apoptotic cells was performed using Fiji software (left). P53 expression levels in the acromegaly model and WT larvae (right). Data are expressed as the mean ± SE (*n* = 3). Statistical difference (*t*-test, *P* < 0.05) is denoted by an asterisk.

To study the effect of GH excess on DNA damage, we retrieved differentially expressed genes (DEGs) between WT and acromegaly zebrafish (one-year-old) following RNA-Seq analysis from different organs and assessed the X2K subnetwork [14] of upregulated genes in the liver, kidney, brain, and larvae to determine the main kinase and transcription factors driving the expression of DEGs. Protein kinases are master regulators of cell function, as they are responsible for the phosphorylation of other proteins; thus, they play an important role in controlling protein function, cellular machine regulation, and information transfer through cell signaling pathways [23]. The X2K results demonstrated that Ataxia-telangiectasia mutated (ATM) and ataxia-telangiectasia and Rad3-related (ATR) (DNA damage sensors) are among the main kinases that drive the expression of upregulated genes (Table 1). These results were confirmed by whole-mount immunostaining using H2A.X antibody (DNA damage marker) in the acromegaly model larvae. The number of H2A.X nuclear-stained cells was significantly high in acromegaly larvae (Figure 3A,B). For quantification of the number of DNA-damaged cells in adult acromegaly fish (one-year-old), we performed tissue disaggregation from the muscle and brain, as described in the materials and methods, and used FACS analysis for the isolation of live cells after the exclusion of Propidium iodide-positive dead cells. Then, we performed H2A.X Immunostaining of FACS-isolated live cells from the brain and muscle. Quantification of H2A.X nuclear-stained cells showed a significant increase of the DNA-damaged cells in acromegaly fish versus WT control (one-year-old) (Figure 3C). Additionally, the heatmap from RNA-Seq data analysis revealed an induction of DNA damage markers [17] in the acromegaly embryo, brain, and kidney (Figure 4A–C). Using GSEA, we found that the induction of DNA damage markers was significant (*p*-value < 0.05, FDR < 0.25) (Figure 4D–F, Table S3) in the acromegaly embryos, brain, and kidney. Taken together, these observations suggest that GH excess in acromegaly model zebrafish is accompanied by an increase in DNA damage in different organs.

### 3.3. Impairment of DNA Repair Pathways in Acromegaly Zebrafish Liver and Induction of Apoptosis in Acromegaly Larvae

**Figure 5:**
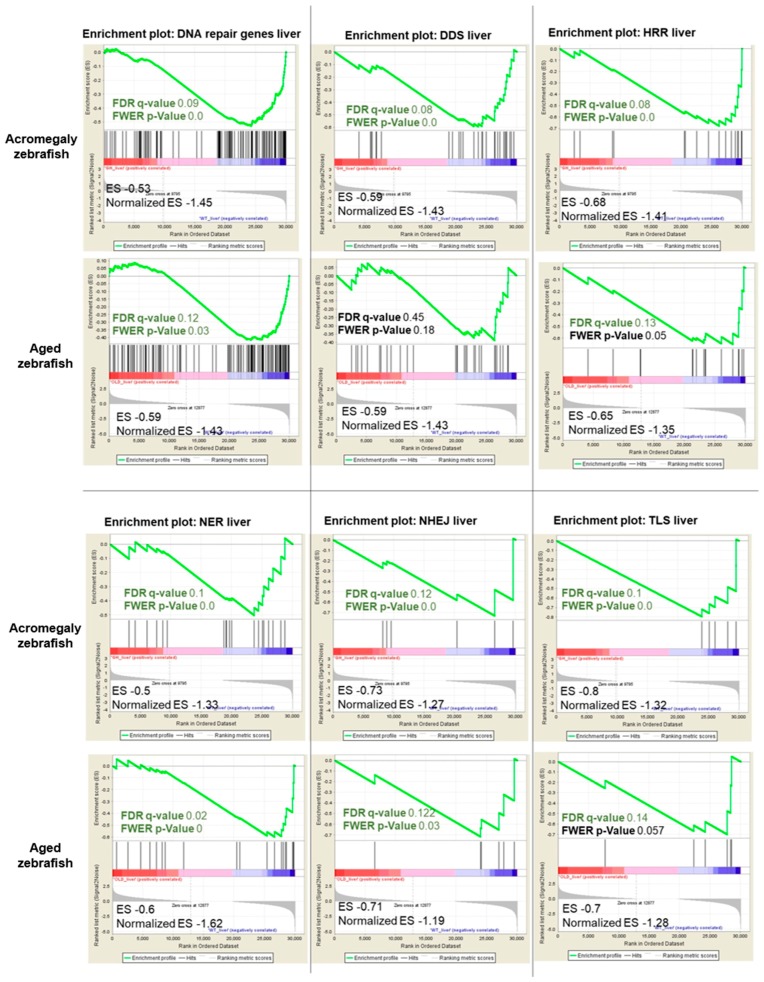
Acromegaly and aged zebrafish impaired DNA repair pathways in the liver. The results of GSEA in acromegaly (one-year-old) and aged zebrafish (three-years-old) liver versus control (one-year-old) showing the familywise-error rate (FWER) *p*-values, FDR, ES, and normalized ES of DNA repair pathways. Significant *p*-value < 0.05 and FDR *q*-value < 0.25 are written in red. According to GSEA [15], the reported *p*-value of 0.0 indicates an actual *p*-value of less than 0.01.

Since DNA repair pathways are fundamental for repairing DNA damage, we investigated whether GH excess also impairs DNA repair. We retrieved a set of genes involved in DNA repair activities in humans [16] and converted them to their zebrafish orthologues using BioMart. Then, we performed GSEA from the WT (one-year-old), acromegaly model (one-year-old), and aged zebrafish (three-year-old) RNA-Seq data, and we found that DNA repair genes were significantly reduced (*p*-value < 0.05, FDR < 0.25) in the liver of acromegaly and aged fish (Figure 5). To better dissect which DNA repair pathway was perturbed due to GH excess, we compared the expression of the genes encoding proteins involved in each of the five main DNA repair pathways: DNA damage response signaling (DDRS), homologous recombination repair (HRR), non-homologous end joining (NHEJ), nucleotide excision repair (NER), and translesion synthesis (TLS). Interestingly, the impairment of DNA repair pathways was even more prominent in the model than in aged zebrafish (Figure 5). All repair pathways were significantly downregulated in the acromegaly zebrafish liver (Figure 5), while downregulation of DNA repair pathways in other organs was not statistically significant (data not shown); however, three out of the five pathways were reduced in the aged zebrafish liver (Figure 5). 

The defect in various DNA repair pathways and DNA damage can activate both membrane death receptors and the endogenous mitochondrial damage pathway, leading to cell death via apoptosis [24]. Consistent with this, we observed a progressive increase in the acridine orange-stained apoptotic cell number in acromegaly larvae with the induction of p53 gene expression (Figure 4E).

In general, these results suggest that GH excess affected cellular protection through the reduction of DNA repair pathways in the liver, the induction of DNA damage, and apoptosis.

## 4. Discussion

Zebrafish are ideal models for studying human diseases owing to their physiological similarity to mammals, ease of genetic manipulations, the optical clarity of embryos and larvae, rapid development and productivity, and cheap housing costs. Moreover, zebrafish models have been developed for many genetic diseases, including embryonic, developmental, carcinogenesis, and immunological diseases, among others [25]. That prompted us to study an acromegaly zebrafish model to study acromegaly pathogenies.

In agreement with our results, previous reports have shown that peripheral lymphocytes of acromegaly patients increased the micronucleus (MN) and oxidative DNA damage [26,27]. Additionally, bone marrow cells isolated from transgenic growth hormone (Tg) mice, showed significantly greater constitutive levels of H2AX foci and chromosomal aberrations [28]. Moreover, the rapid growth of GH-transgenic salmon was associated with substantial telomere loss per time unit, which may be due to elevated oxidative damage to DNA [29].

Acromegaly patients, GH transgenic rats, and GH-transgenic salmon showed a significant induction of oxidative stress [26,29,30]. Since oxidative stress is known to cause DNA damage involving point mutations because of base oxidation, the elevation of oxidative stress could provide a reasonable explanation of increased DNA damage in acromegaly patients [26,29].

Growth hormone secretion occurs in an intermittent or pulsatile pattern from the pituitary gland according to body needs rather than through continuous release [31]. However, this pulsatile pattern is lost in acromegaly due to continuous GH hypersecretion [32]. In acromegaly patients, the basal GH level can increase up to 100-fold [33].

In this study, endogenous growth hormone was only detected in the brain of WT and acromegaly adult zebrafish as the pituitary gland is the source of GH production [34]. Endogenous GH was significantly reduced in acromegaly zebrafish larvae and adults, probably due to the negative feedback loop [20].

On the other hand, exogenous GH gene expression was detected by RT-PCR in acromegaly fish at the larvae and adult stage, but not in WT fish. Acromegaly liver and muscle showed strong tilapia GH gene expression, which indicates that the EF1 promoter drives GH gene expression in the adult stage as well; however, it was barely detected in the brain. These results are consistent with previous studies which showed that the EF1 promoter encounters a progressive inactivation in the nervous system [35,36].

The major GH effects are mediated by the GH/IGF-1 axis, which is the key controller of somatic growth [37,38]. IGF-1 is secreted mainly from the liver to mediate most of the GH physiological effect [21]. In agreement with this, our acromegaly model showed a significant increase in liver IGF-1. However, muscle did not show a significant induction of the IGF-1 mRNA level.

Unlike the pulsatile nature of GH secretion, the IGF-1 level is much more constant throughout the day, thus, the IGF-1 level is being used more frequently for the routine diagnosis of acromegaly [1,4]. According to age, the normal serum IGF-1 level can range from 69–483 ng/mL [39]. In acromegaly patients, the average serum IGF-1 level is about 676 ng/mL and may reach 1000 ng/mL [33], meaning that it can increase from approximately 1.5-fold to 10-fold. Our acromegaly model showed a four-fold increase in liver IGF-1 mRNA level and a five-fold increase in IGF-1 mRNA level at the larval stage, indicating that our acromegaly model IGF-1 level may resemble the acromegaly IGF-1 level.

Elevation of the GH/IGF-1 axis in our acromegaly model was associated with significant down-regulation of DNA repair pathways. Consistent with these findings, GH/IGF-1 deficiency in primary fibroblasts derived from dwarf rats elevated cellular DNA repair capacity [40].

The recommended medical therapies for acromegaly according to the guidelines of the Endocrine Society (ENDO) are pegvisomant (GH-receptor blocking agents) and somatostatin receptor ligands (SRLs) [41], which are dependent on the blocking GH from acting. However, a better understanding of acromegaly perturbed health parameters may help in the identification of additional potential targets for therapeutic interventions. Here, we produced an acromegaly zebrafish model and investigated how GH excess functions in an acromegaly model, focusing on cellular integrity. Cellular integrity is a fundamental parameter that reflects the health status of an organism. The induction of DNA damage and decline of DNA repair in an acromegaly zebrafish model provide a better understanding of acromegaly and can be used as a marker for the diagnosis and follow-up of acromegaly patients.

X2K analysis revealed that ATM and ATR (DNA damage sensors) are among the main protein kinases that drive the expression of acromegaly zebrafish upregulated genes; however, other protein kinases were enriched as well (Table 1), suggesting the perturbation of additional signaling pathways in the acromegaly model.

## 5. Conclusions

We produced an acromegaly zebrafish model that overexpresses tilapia GH and the red fluorescence protein (RFP) reporter gene and performed RNA-Seq data analysis from different organs using various approaches to identify the perturbed biological processes. RNA-Seq data analysis suggested the impairment of DNA repair pathways in the liver and elevation of DNA damage in different organs. DNA damage was confirmed by immunostaining experiments, thus reinforcing the previous notion that blood lymphocytes from acromegalic patients exhibit an elevation of DNA damage [26,27]. However, our data suggest that GH excess elevates DNA damage in various organs.

One of the main health complications associated with acromegaly is the increased incidence of colon cancer [1]. Since genomic instability and increased DNA damage are among the main causes of cancer induction [42], the elevation of DNA damage in our model highlights the potential role of DNA damage in colorectal cancer induction in acromegaly. Consistently, previous studies suggested that the positive association between micronucleus (MN) DNA damage frequency and serum IGF-1 levels may predict the risk of malignancy in acromegalic patients [26].

## Figures and Tables

**Table 1:** X2K analysis of upregulated genes in acromegaly zebrafish model.

Kinases	Substrate in Input	Substrates in Database	Combined Score
**The main eight kinases regulate Upregulated genes in Acromegaly brain**			
HIPK2	9	43	32.7
MAPK14	9	43	32.72258
PRKDC	16	461	15.36258
ATR	11	227	15.34714
TAF1	6	63	14.71918
CSNK2A1	5	42	13.43954
CHEK2	17	521	13.2443
CDK2	5	41	12.59273
**The main eight kinases regulate Upregulated genes in Acromegaly liver**			
MAPK1	34	312	76.13761
CSNK2A1	38	521	57.07884
GSK3B	36	600	41.68791
CHUK	14	90	35.26104
MAPK14	29	461	34.38588
ATM	18	177	34.03207
CSNK2A2	22	267	33.89444
AKT1	21	256	31.73479
**The main eight kinases regulate Upregulated genes in Acromegaly Kidney**			
MAPK1	17	312	36.51985
CSNK2A1	21	521	33.22942
CSNK2A2	15	267	31.16424
MAPK8	14	275	26.28329
ATM	11	177	24.1775
HIPK2	7	43	23.41801
MAPK14	17	461	22.83626
ATR	7	63	18.45281
**The main eight kinases regulate Upregulated genes in Acromegaly larvae**			
MAPK1	28	312	53.17025
AKT1	22	256	41.05688
CSNK2A1	32	521	39.35772
MAPK3	21	251	34.32238
ATM	17	177	30.62717
HIPK2	10	43	28.47911
ATR	11	63	26.07581
PRKCD	14	146	24.01391

ATM and ATR (DNA damage sensors) are among the main protein kinases that drive the expression of acromegaly zebrafish upregulated genes. X2K created from upregulated genes of RNA-Seq data in the acromegaly zebrafish (one-year-old) compared to wild-type (WT) showing the main eight kinase proteins that regulate gene expression changes in the brain, kidney, and liver, in addition to larvae (3 dpf).

## Supplementary Materials

The following are available online at http://www.mdpi.com/2079-7737/7/4/47/s1, Table S1: Primers used for PCR, Table S2: Sequences of qPCR primers for selected genes, Table S3: DNA damage gene set used for GSEA.
