# Supplementary material for: A Zebrafish Acromegaly Model Elevates DNA Damage and Impairs DNA Repair Pathways
Source: Biology (Basel). 2018 Oct 17;7(4):47. doi: 10.3390/biology7040047 (PMC6315448; doi:10.3390/biology7040047)
Supplement: Supplementary file 1 [file biology-07-00047-s001.pdf]

biology

MDPI

Article

A Zebrafish Acromegaly Model Elevates DNA Damage and Impairs DNA Repair Pathways

Abdalla Elbialy [1], Shuichi Asakawa [1], Shugo Watabe [2] and Shigeharu Kinoshita [1,*]

Supplementary Tables

Table S1. Primers used for PCR.

Primer	Sequence	Reference
GH. F	ATCAGAACCACCGACTCACATCATAATC	This study
GH. R	CAATGCAACACATTTATTTCACAGAATAACATTATTCC	This study

Table S2. Sequences of qPCR primers for selected genes.

Primer	Forward Primer (5 to 3)	Reverse Primer (5 to 3)	Reference
Insulin-like growth factor 1 (IGF1a)	ACACAGGGGGCAGAAACTAT	AAGATGGGGCTTAAACGTCC	[42]
Insulin-like growth factor 3 (IGF1b)	CGTGATGTCCCTGCATCTGT	TTCTGGTATCGCCGCTGAAA	This study
Zebrafish growth hormone (GHz)	AGGTCTTATGCCTGAGGAACG	AAGGTCTGGCTGGGAAACTC	[42]
Elongation factor 1 alpha (EF1)	TGTCCTCAAGCCTGGTATGG	TGGGTCGTTCTTGCTGTCTC	[42]
Tumor suppressor p53 (p53)	ATCATCTGAGCCCAAACAGG	AAATGACCCCTGTGACAAGC	[17]

Table S3. DNA damage gene set used for GSEA.

Zebrafish Gene Name	Gene Symbol
X-ray repair complementing defective repair in Chinese hamster cells 2	xrcc2
UDP glucuronosyltransferase 1 family a, b	utg1ab
uracil-DNA glycosylase a	unga
RAD50 homolog	rad50
RAD23 homolog Aa	rad23aa
excision repair cross-complementing rodent repair deficiency, complementation group 1	ercc1
excision repair cross-complementing rodent repair deficiency, complementation group 3	ercc3
damage specific DNA binding protein 1	ddb1
protein kinase Chk2	chk2
ataxia telangiectasia mutated	atm
